# Association of Lactate Dehydrogenase with In-Hospital Mortality in Patients with Acute Aortic Dissection: A Retrospective Observational Study

**DOI:** 10.1155/2020/1347165

**Published:** 2020-01-07

**Authors:** Huaping He, Xiangping Chai, Yang Zhou, Xiaogao Pan, Guifang Yang

**Affiliations:** ^1^Department of Emergency Medicine, The Second Xiangya Hospital, Central South University, Changsha, China; ^2^Emergency Medicine and Difficult Diseases Institute, Central South University, Changsha, China

## Abstract

**Background:**

Evidence regarding the relationship between serum lactate dehydrogenase (LDH) levels and in-hospital mortality in acute aortic dissection (AAD) patients is extremely limited. We aimed to investigate the relationship between LDH and in-hospital mortality in AAD patients.

**Methods:**

The present study was a retrospective observational study. A total of 1526 participants with acute aortic dissection were involved in a hospital in China from January 2014 to December 2018. The target-independent variable was LDH measured at baseline, and the dependent was all-cause mortality during hospitalization. Covariates involved in this study included age, gender, body mass index (BMI), hypertension, diabetes, smoking, stroke, atherosclerosis, systolic blood pressure (SBP), diastolic blood pressure (DBP), white blood cell (WBC), hemoglobin (Hb), alanine transaminase (ALT), aspartate aminotransferase (AST), albumin (ALB), creatinine (Cr), symptom, type of AAD (Stanford), and management.

**Results:**

The average age of 1526 selected participants was 52.72 ± 11.94 years old, and about 80.41% of them were male. The result of the fully adjusted model showed LDH was positively associated with in-hospital mortality in AAD patients after adjusting confounders (OR = 1.09, 95% CI 1.05 to 1.13). A nonlinear relationship was detected between LDH and in-hospital mortality in AAD patients after adjusting for potential confounders (age, gender, BMI, hypertension, diabetes, stroke, atherosclerosis, smoking, symptom, SBP, DBP, WBC, Hb, ALT, AST, ALB, Cr, type of AAD (Stanford), and management), whose point was 557. The effect sizes and the confidence intervals of the left and right sides of the inflection point were 0.90 (0.74–1.10) and 1.12 (1.06–1.19), respectively. Subgroup analysis in participants showed that the relationship between LDH and in-hospital mortality was stable, and all of the *P* value for the interaction in different subgroup were more than 0.05.

**Conclusions:**

The relationship between LDH and in-hospital mortality in AAD patients is nonlinear. LDH was positively related with in-hospital mortality when LDH is more than 557.

## 1. Introduction

Acute aortic dissection (AAD) is a catastrophic aortic disease with high mortality and morbidity that requires immediate diagnosis and treatment [1, 2]. From 1% to 2% of patients with AAD die per hour for the first 24∼48 hours [3]. Previous studies have found that many noninvasive markers, such as CRP and D-dimer, are considered to be associated with AAD severity [4, 5]. As a cytoplasmic enzyme, LDH is widely expressed in tissues. Increased LDH levels can be caused in many conditions such as malignancies, tissue injury, hypoxia, necrosis, and hemolysis [6]. Studies have shown that in the case of idiopathic pulmonary arterial hypertension, patients with high levels of LDH had a low cumulative survival rate [7]. In addition, Lu et al. [8] have demonstrated that in patients with sepsis, LDH levels increased during hospitalization also indicated a worse short-term prognosis. Moreover, increased LDH levels are also associated with worse cardiovascular mortality in the arsenic-endemic areas of southwestern Taiwan [9]. However, there are little available data on the relationship between LDH and clinical outcomes in AAD patients.

## 2. Participants and Methods

### 2.1. Study Design and Settings

This is a retrospective observational study design. Medical records of AAD patients admitted to the Second Xiangya Hospital of Central South University from January 2014 to December 2018 were investigated. The study was reviewed and approved by the hospital ethics committee, and as a retrospective study, informed consent was waived.

The type of AAD was classified according to Stanford criteria, and the diagnosis of AAD was confirmed by computed tomography angiography (CTA) or magnetic resonance angiography (MRA) [10]. AAD patients with a time interval of ≤14 days from the onset of symptoms to hospital admission were included in the present study. Exclusion criteria included (1) uncompleted LDH tests, (2) prior history of a malignant tumor or liver cirrhosis, (3) diagnosis with pregnancy, and (4) presence of AAD for more than 14 days.

### 2.2. AAD Treatment

For AAD patients combined with high blood pressure, urapidil, sodium nitroprusside, or nitroglycerin is administered intravenously to reduce systolic blood pressure (SBP) to 100–120 mmHg. All patients were given beta-blockers except for contraindications. Acute type A aortic dissection patients and a small portion of acute type B aortic dissection patients were surgically repaired under cardiopulmonary bypass. Under general anesthesia, patients with acute type B aortic dissection were performed an endovascular repair using available grafts. The criteria for endovascular treatment of type B AAD were based on the 2014 ESC guidelines. Thoracic endovascular aortic repair (TEVAR) is the treatment of choice in complicated acute type B AD. The term “complicated” means persistent or recurrent pain, uncontrolled hypertension despite full medication, early aortic expansion, malperfusion, and signs of rupture (haemothorax, increasing periaortic, and mediastinal hematoma). AAD patients who did not undergo surgery were given conservative medical treatment [11].

### 2.3. Variables Included for Analysis

Serum LDH was measured at baseline for all the AAD participants. A plasma LDH assay was performed on blood collected with a Roche Cobas automated platform, using a colorimetric pyruvate-lactate enzymatic assay technique. Sample collection always made before any surgical/endovascular treatment. The normal reference range is 109 to 245 *μ*/L at our hospital. All LDH measures were performed at our hospitals' clinical laboratory. The covariates used in this study can be classified as follows: (1) demographic data; (2) variables that can affect LDH or in-hospital mortality among AAD patients reported by previous literature; and (3) based on our clinical experiences. The following clinical data were collected on admission: age, gender, body mass index (BMI), hypertension, diabetes, smoking, stroke, atherosclerosis, systolic blood pressure (SBP), diastolic blood pressure (DBP), white blood cell (WBC), hemoglobin (Hb), alanine transaminase (ALT), aspartate aminotransferase (AST), albumin (ALB), creatinine (Cr), symptom, and type of AAD (Stanford). Then, we collected the data of management in AAD patients. No multiple imputation was performed in this study because the missing for the covariates was less than 5% [12].

### 2.4. Statistical Analysis

We followed the methods of Chen et al. [13] in this study. R (http://www.R-project.org) and Empower States (http://www.empowerstates.com, X&Y Solution, Inc, Boston, MA) software were used to carry out all the statistical analyses. Statistical data were presented in mean ± standard deviation for normal data while non-normal data, interquartile range (IQR), and median were used. The categorical variables were presented as percentages and numbers. Wilcoxon Mann–Whitney tests for non-normally distributed continuous variables and unpaired Student t-tests for normally distributed continuous variables were used to establish the correlations among the survivor and the nonsurvivor groups. We used chi-squared test (categorical variables), one-way ANOVA test (normal distribution), or one-way ANOVA test (skewed distribution) to test for differences among different LDH groups (Tertile). Three criteria were used in the process of this data analysis. First, what is the relationship between LDH and in-hospital mortality (linear or nonlinear)? Second,which factors interfere or modify the relationship between LDH and in-hospital mortality. Third, what is the true relationship between LDH and in-hospital mortality while adjusting the interference factors or after the stratified analysis? Therefore, univariate and multivariate analyses were employed firstly. We constructed three models: crude model, with no adjustment of covariates; model I, adjusted for sociodemographic data; and model II, model I including other covariates presented in Table 1. A sensitivity analysis was carried out for robustness during data analysis. LDH was converted to an absolute variable, and *P* value for tendency was determined. And then, to address for nonlinearity of LDH and in-hospital mortality, a generalized additive model and smooth curve fitting (penalized spline method) were conducted. If nonlinearity was detected, we first calculated the inflection point using recursive algorithm and then constructed a two-piecewise on both sides of the inflection point. How to determine which model is more suitable for fitting the correlation between target independent variable, and the dependent variable was mainly based on the *P* value of the log-likelihood ratio test. Survival curves were constructed using the Kaplan–Meier method estimates and compared with the log-rank test. Finally, the subgroup analyses were performed using stratified models. For a continuous variable, we first converted it to a categorical variable according to the clinical cut point or tertile and then performed an interaction test. Tests for effect modification for those of subgroup indicators were followed by the likelihood ratio test.

## 3. Results

### 3.1. Baseline Characteristics of Selected Participants

**Figure 1:**
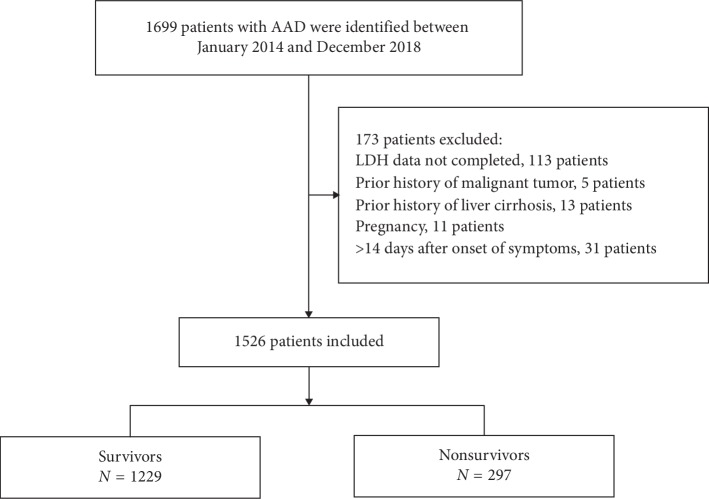
Flow chart of patient enrollment.

A total of 1526 patients with AAD were enrolled in the present study based on the inclusion and exclusion criteria (see Figure 1 for a flow chart). Patient characteristics are shown in Table 1 according to the tertile of LDH. There were no significant differences in age, gender, hypertension, diabetes, smoking, and stroke among these groups and the level of SBP, DBP, Hb, and ALB. Participants with the highest group of LDH (T3) had higher values in BMI, a symptom of chest pain, abdominal pain, and syncope, WBC, ALT, AST, Cr, A type of AAD, and management of medical and surgical than those of other groups.

### 3.2. Univariate Analysis

We listed the results of univariate analyses in Table 2. By univariate analysis, we found that age, BMI, gender, hypertension, diabetes, stroke, atherosclerosis, back pain, and other symptom were not associated with in-hospital mortality. We also found that SBP (0.99, 0.98-0.99), DBP (0.98, 0.97-0.98), Hb (0.99, 0.99-1.00), ALB (0.96, 0.94–0.99), smoking (0.74, 0.56–0.99), abdominal pain (0.49, 0.27–0.91), B type of AAD (Stanford) (0.16, 0.12–0.22), endovascular (0.02, 0.01–0.03), and surgical (0.08, 0.05–0.11) were negatively associated with in-hospital mortality. In contrast, univariate analysis showed that WBC (1.07, 1.04–1.11), ALT (1.00, 1.00-1.00), AST (1.00, 1.00-1.00), Cr (1.00, 1.00-1.00), smoking (0.74, 0.56–0.99), syncope (2.87, 1.14–7.20), and LDH (1.10, 1.06–1.14) (per 100 increment) were positively correlated with in-hospital mortality.

### 3.3. Results of Unadjusted and Adjusted

In this study, we constructed three models to analyze the independent effects of LDH on in-hospital mortality (univariate and multivariate). The effect sizes (OR) and 95% confidence intervals were listed in Table 3. In the unadjusted model (crude model). The model-based effect size can be explained as the difference in 100 u/L of LDH associated with in-hospital mortality (1.10, 95% CI 1.06–1.14). In the minimum-adjusted model (model I), the LDH was increased by 100 *μ*/L, in-hospital mortality increased by 10% (1.10, 95% CI 1.06–1.14). In the fully adjusted model (model II) (adjusted all covariates presented in Table 1) for each additional 100 *μ*/L of LDH, in-hospital mortality increased by 9% (1.09, 95% CI 1.05–1.13). For the purpose of sensitivity analysis, we converted the LDH from continuous variable to categorical variable (tertile of LDH); the *P* for trend of LDH with categorical variables in the fully adjusted model was not consistent with the result when LDH is a continuous variable. However, we found the trend of the effect size in different LDH groups was consistent with the result when LDH is a continuous variable.

### 3.4. The Results of Nonlinearity of LDH and In-Hospital Mortality

**Figure 2:**
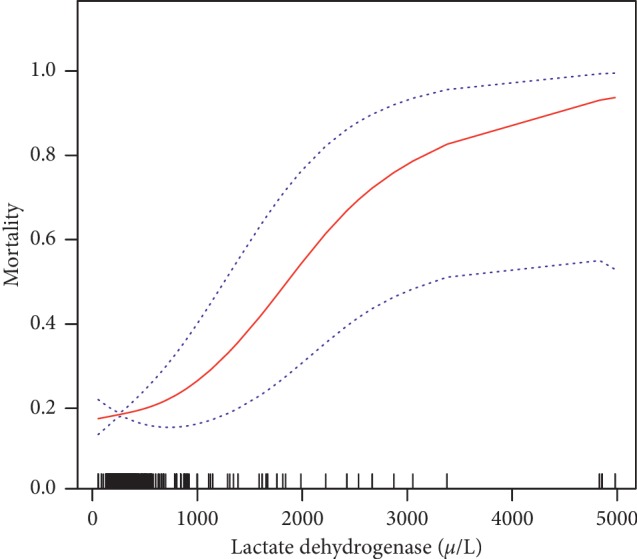
Association between lactate dehydrogenase and in-hospital mortality. A nonlinear association between lactate dehydrogenase and in-hospital mortality was found in a generalized additive model (GAM). The solid red line represents the smooth curve fit between variables. Blue bands represent the 95% confidence interval from the fit. All adjusted for age, gender, BMI, hypertension, diabetes, stroke, atherosclerosis, smoking, symptom, systolic blood pressure, diastolic blood pressure, type of AAD (Stanford), admission of white blood cell, hemoglobin, alanine transaminase, aspartate aminotransferase, albumin, creatinine, and management.

In the present study, we analyzed the nonlinear relationship between LDH and in-hospital mortality (Figure 2). Smooth curve and the result of generalized additive model showed that the relationship between LDH and in-hospital mortality was nonlinear after adjusting for age, gender, BMI, hypertension, diabetes, stroke, atherosclerosis, smoking, symptom, systolic blood pressure, diastolic blood pressure, type of AAD (Stanford), admission of white blood cell, hemoglobin, alanine transaminase, aspartate aminotransferase, albumin, creatinine, and management. We used both linear regression model and two-piecewise linear regression model to fit the association and select the best-fit model based on *P* for log-likelihood ratio. Because the *P* for log-likelihood ratio test was less than 0.05, we chose a nonlinear regression model for fitting the association between LDH and in-hospital mortality because it can accurately represent the relationship. By two-piecewise linear regression model and recursive algorithm, we calculated the inflection point was 557. On the left side of inflection point, the effect size and 95% CI were 0.90, 0.74–1.10, respectively. On the right side of inflection point, the effect size and 95% CI were 1.12, 1.06–1.19, respectively (Table 4).

### 3.5. Survival Curve Analysis

**Figure 3:**
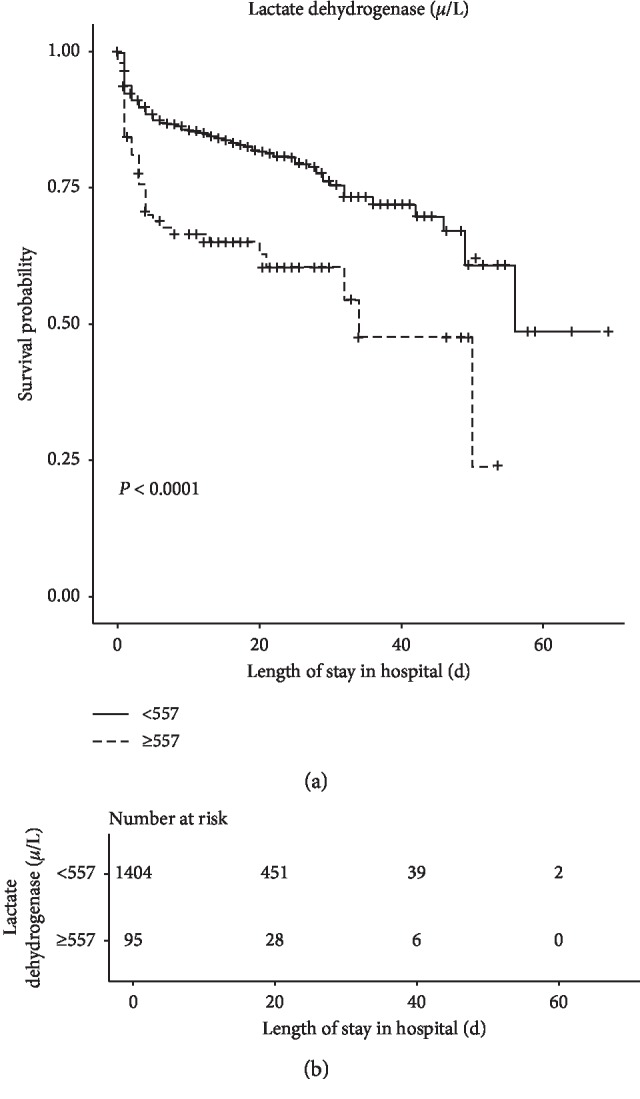
Survival curve analysis in the chronological trend in mortality after AAD.

Kaplan–Meier analysis showed that the cumulative in-hospital survival rate was significantly lower in high-level LDH group (*P*=0.013) (Figure 3).

### 3.6. Subgroup Analysis

We used age, BMI, SBP, DBP, WBC, Hb, ALT, AST, ALB, Cr, gender, symptom, hypertension, diabetes, smoking, stroke, atherosclerosis, type of AAD (Stanford), and management as the stratification variables to observe the trend of effect sizes in these variables (Table 5) We noted that none interactions were observed based on our a priori specification (all *P* values for interaction >0.05).

## 4. Discussion

The major finding of this study was that LDH levels were positively associated with in-hospital mortality in AAD patients after adjusting other covariates. Besides, we also find the trend of the effect sizes on the left and right sides of the inflection point is not consistent [left 0.90 (95% CI 0.74–1.10) and right 1.12 (95% CI 1.06–1.19)]. This result suggests a threshold effect on the independent association between LDH and in-hospital mortality in AAD patients. Subgroup analysis will help us to better understand the trend of LDH and in-hospital mortality in different populations. The results of this study find the association between LDH and in-hospital mortality was stable, and all of the *P* value for the interaction in different subgroups were more than 0.05.

Mortality prediction in AAD is clinically important. Previous studies have shown that organ malperfusion and hemodynamic statuses, such as hypotension, shock, cardiac tamponade, pulse deficits, and kidney failure, confer an even higher mortality rate in AAD patients. Morello et al. [14] demonstrated that plasma lactate dehydrogenase levels could predict mortality in acute aortic syndromes. However, in their research, they mainly discussed AAS and did not focus on aortic dissection. We know that AAS includes classic acute aortic dissection (AAD), intramural hematoma, and penetrating atherosclerotic aortic ulcer. In addition, they did not discuss the nonlinear relationship between LDH and in-hospital mortality in AAD patients. Furthermore, there were only 166 AAD patients in their research, which were far less than our study.

Increased LDH levels can be caused in many conditions such as malignancies, tissue injury, hypoxia, necrosis, and hemolysis [6]. Previous studies have demonstrated that myocardium, skeletal muscle, liver, red blood cells, and intestinal tract are the most well-known tissue sources of LDH [15]. Therefore, these tissue damage or ischemia are potential sources of elevated plasma LDH levels in AAD patients. When the oxygen supply is insufficient, the enzyme converts the final product of glycolysis, pyruvate, to lactic acid. It enables hypoxic cells to produce adenosine triphosphate and remain viable in a relatively low oxygen environment [16].

Elevated levels of LDH are not only associated with hypoxia but also a marker of inflammation and oxidative stress, which indicated an increased risk of AAD and a poor prognosis. In the pathogenesis and progression of AAD, the role of hypoxia and inflammation is well defined. The research of Colgan et al. [17] showed that hypoxia can induce lactate dehydrogenase expression. Moreover, Gaisl et al. [18] have found that the relationship between hypoxia and aortic dissection may be intermittent hypoxia associated with autonomic nervous system activation and consequently increased oxidative stress. In addition, Drent et al. [19] concluded that LDH is an indicator of pathological conditions in the lungs, such as cell damage or inflammation. Furthermore, Duan et al. [20] deduced that inflammation is related to preoperative hypoxemia in patients with acute Stanford type A aortic dissection. Overall, the mechanism of elevated lactate dehydrogenase in patients with AAD may be due to hypoxia or inflammatory reactions.

The clinical value of this study is as follows: (1) Elevated LDH may be used by physicians for counseling patients and their families in helping them to understand their predicted risk and to have realistic expectations in terms of outcomes, especially in those deemed to be at high risk for in-hospital mortality; and (2) the findings of this study should be helpful for future research on the establishment of diagnostic or predictive models of in-hospital mortality in AAD patients.

Our study has some strengths: (1) our sample size is relatively large; (2) we address the nonlinearity in the present study and further explore this; (3) this study is an observational study and therefore susceptible to potential confounding, and we used strict statistical adjustment to minimize residual confounders; and (4) we handled target independent variables as both continuous variable and categorical variables. Such an approach can reduce the contingency in the data analysis and enhance the robustness of results.

There are some limitations in this study. First, the study only discussed the relationship between LDH and in-hospital mortality but did not discuss the relationship between LDH and the long-term prognosis of AAD patients, which is the direction of our future research. Second, our findings are based on a Chinese patient; it is unclear whether studies of other nationalities will yield similar findings. In western countries and in Japan, the average age of patients with AADs is way higher (median 65–70 years). Hence, mortality prediction models extrapolated from younger Chinese populations may have limited external validity. Finally, as a single-center study, the results must be interpreted with caution when extrapolating them into other settings.

## 5. Conclusions

The relationship between LDH and in-hospital mortality in AAD patients is nonlinear. LDH was positively related with in-hospital mortality when LDH is more than 557.

## Figures and Tables

**Table 1:** Baseline characteristics of the patients (*N*=1526).

Characteristic	Lactate dehydrogenase (*μ*/L) (tertile)				*P* value
	Total	T1 (54.6–209.3)	T2 (209.7–274.7)	T3 (274.8–4981.0)	
No. of patients	1526	504	511	511	
Age (years, mean ± sd)	52.72 ± 11.94	53.71 ± 11.87	52.52 ± 11.82	51.94 ± 12.09	0.055
BMI (kg/m^2^, mean ± sd)	25.03 ± 4.28	24.44 ± 3.94	25.20 ± 4.33	25.44 ± 4.49	<0.001
Gender					0.238
Male	1227 (80.41%)	397 (78.77%)	407 (79.65%)	423 (82.78%)	
Female	299 (19.59%)	107 (21.23%)	104 (20.35%)	88 (17.22%)	
Hypertension					0.362
No	469 (30.73%)	162 (32.14%)	162 (31.70%)	145 (28.38%)	
Yes	1057 (69.27%)	342 (67.86%)	349 (68.30%)	366 (71.62%)	
Diabetes					0.386
No	1468 (96.20%)	480 (95.24%)	494 (96.67%)	494 (96.67%)	
Yes	58 (3.80%)	24 (4.76%)	17 (3.33%)	17 (3.33%)	
Smoking					0.501
No	1044 (68.41%)	337 (66.87%)	359 (70.25%)	348 (68.10%)	
Yes	482 (31.59%)	167 (33.13%)	152 (29.75%)	163 (31.90%)	
Stroke					0.865
No	1465 (96.00%)	482 (95.63%)	491 (96.09%)	492 (96.28%)	
Yes	61 (4.00%)	22 (4.37%)	20 (3.91%)	19 (3.72%)	
Atherosclerosis					0.002
No	1393 (91.28%)	443 (87.90%)	470 (91.98%)	480 (93.93%)	
Yes	133 (8.72%)	61 (12.10%)	41 (8.02%)	31 (6.07%)	
Symptom					0.002
Chest pain	1166 (76.41%)	362 (71.83%)	401 (78.47%)	403 (78.86%)	
Back pain	69 (4.52%)	27 (5.36%)	26 (5.09%)	16 (3.13%)	
Abdominal pain	108 (7.08%)	37 (7.34%)	29 (5.68%)	42 (8.22%)	
Syncope	19 (1.25%)	5 (0.99%)	3 (0.59%)	11 (2.15%)	
Other	164 (10.75%)	73 (14.48%)	52 (10.18%)	39 (7.63%)	
Type of AAD (Stanford)					<0.001
A	703 (46.07%)	200 (39.68%)	221 (43.25%)	282 (55.19%)	
B	823 (53.93%)	304 (60.32%)	290 (56.75%)	229 (44.81%)	
SBP (mmHg, mean ± sd)	145.97 ± 29.23	144.38 ± 27.12	147.46 ± 28.13	146.05 ± 32.15	0.242
DBP (mmHg, mean ± sd)	81.79 ± 18.14	81.10 ± 16.21	82.98 ± 17.82	81.28 ± 20.14	0.189
WBC (×10^9^/L, mean ± sd)	11.21 ± 4.15	9.79 ± 3.50	11.28 ± 3.81	12.55 ± 4.59	<0.001
Hb (g/L, mean ± sd)	124.96 ± 20.83	123.54 ± 20.72	125.94 ± 20.38	125.40 ± 21.34	0.158
ALT (*μ*/L, mean ± sd)	78.85 ± 422.73	22.44 ± 17.32	33.49 ± 41.04	179.85 ± 718.99	<0.001
AST (*μ*/L, mean ± sd)	100.92 ± 639.72	22.16 ± 16.13	28.49 ± 27.54	251.03 ± 1090.29	<0.001
ALB (g/L, mean ± sd)	35.61 ± 4.62	35.49 ± 4.48	35.81 ± 4.65	35.53 ± 4.72	0.483
Cr (*μ*mol/L, mean ± sd)	116.16 ± 135.88	109.73 ± 146.47	101.39 ± 95.58	137.28 ± 155.59	<0.001
Management					<0.001
Medical	412 (27.00%)	130 (25.79%)	118 (23.09%)	164 (32.09%)	
Endovascular	647 (42.40%)	236 (46.83%)	230 (45.01%)	181 (35.42%)	
Surgical	467 (30.60%)	138 (27.38%)	163 (31.90%)	166 (32.49%)	
Mortality					<0.001
Survivor	1229 (80.54%)	423 (83.93%)	429 (83.95%)	377 (73.78%)	
Nonsurvivor	297 (19.46%)	81 (16.07%)	82 (16.05%)	134 (26.22%)	
Length of stay in hospital (d)	16.06 ± 10.11	16.55 ± 9.72	16.36 ± 9.34	15.27 ± 11.15	0.097
Length of stay in ICU (d)	2.18 ± 5.08	1.78 ± 4.55	2.14 ± 5.20	2.56 ± 5.38	0.221

Abbreviations: BMI, body mass index; SBP, systolic blood pressure; DBP, diastole blood pressure; WBC, white blood cell; Hb, hemoglobin; ALT, alanine transaminase; AST, aspartate aminotransferase; ALB, albumin; Cr, creatinine; AAD, acute aortic dissection.

**Table 2:** Univariate analysis for in-hospital mortality.

	OR	95 CI	*P* value
Age (years)	1.00	(0.99, 1.01)	0.898
BMI (kg/m^2^)	1.00	(0.97, 1.03)	0.825
SBP (mmHg)	0.99	(0.98, 0.99)	<0.001
DBP (mmHg)	0.98	(0.97, 0.98)	<0.001
WBC (×10^9^/L)	1.07	(1.04, 1.11)	<0.001
Hb (g/L)	0.99	(0.99, 1.00)	0.003
ALT (*μ*/L)	1.00	(1.00, 1.00)	<0.001
AST (*μ*/L)	1.00	(1.00, 1.00)	<0.001
ALB (g/L)	0.96	(0.94, 0.99)	0.005
Cr (*μ*mol/L)	1.00	(1.00, 1.00)	<0.001
Gender			
Male	Ref		
Female	1.02	(0.74, 1.40)	0.896
Hypertension			
No	Ref		
Yes	0.99	(0.75, 1.30)	0.920
Diabetes			
No	Ref		
Yes	0.97	(0.50, 1.89)	0.922
Smoking			
No	Ref		
Yes	0.74	(0.56, 0.99)	0.040
Stroke			
No	Ref		
Yes	1.64	(0.92, 2.90)	0.094
Atherosclerosis			
No	Ref		
Yes	1.17	(0.76, 1.80)	0.476
Symptom			
Chest pain	Ref		
Back pain	1.09	(0.61, 1.97)	0.764
Abdominal pain	0.49	(0.27, 0.91)	0.025
Syncope	2.87	(1.14, 7.20)	0.025
Other	0.74	(0.48, 1.16)	0.187
Type of AAD (Stanford)			
A	Ref		
B	0.16	(0.12, 0.22)	<0.001
Management			
Medical	Ref		
Endovascular	0.02	(0.01, 0.03)	<0.001
Surgical	0.08	(0.05, 0.11)	<0.001
Lactate dehydrogenase (*μ*/L)			
(per 100 increment)	1.10	(1.06, 1.14)	<0.001

Abbreviations: CI, confidence interval; OR, odds ratio; BMI, body mass index; SBP, systolic blood pressure; DBP, diastolic blood pressure; WBC, white blood cell; Hb, hemoglobin; ALT, alanine transaminase; AST, aspartate aminotransferase; ALB, albumin; Cr, creatinine; AAD, acute aortic dissection.

**Table 3:** Relationship between lactate dehydrogenase and in-hospital mortality in different models.

Exposure	Crude model (OR, 95 CI, *P*)	Model I (OR, 95 CI, *P*)	Model II (OR, 95CI, *P*)
Lactate dehydrogenase (*μ*/L) (per 100 increment)	1.10 (1.06, 1.14) <0.001	1.10 (1.06, 1.14) <0.001	1.09 (1.05, 1.13) <0.001
Lactate dehydrogenase (*μ*/L) (tertile)			
T1	Ref	Ref	Ref
T2	1.00 (0.71, 1.40) 0.992	1.00 (0.71, 1.40) 0.998	1.05 (0.65, 1.67) 0.853
T3	1.86 (1.36, 2.53) <0.001	1.86 (1.37, 2.54) <0.001	1.25 (0.79, 1.99) 0.346
*P* for trend	<0.001	<0.001	0.337

Abbreviations: CI, confidence interval; OR, odds ratio. Model I adjusted for age, gender, and BMI. Model II adjusted for age, gender, BMI, hypertension, diabetes, stroke, atherosclerosis, smoking, symptom, systolic blood pressure, diastolic blood pressure, type of AAD (Stanford), admission of white blood cell, hemoglobin, alanine transaminase, aspartate aminotransferase, albumin, creatinine, and management.

**Table 4:** The results of two-piecewise linear model.

	Mortality (OR, 95% CI)	*P* value
Fitting model by standard linear regression (per 100 increment)	1.09 (1.05, 1.13)	<0.001
Fitting model by two-piecewise linear regression (per 100 increments)		
Inflection point of lactate dehydrogenase	557	
≤557	0.90 (0.74, 1.10)	0.296
>557	1.12 (1.06, 1.19)	<0.001
*P* for log-likelihood ratio test	0.048	

Abbreviations: CI, confidence interval; OR, odds ratio. Adjusted: age, gender, BMI, hypertension, diabetes, stroke, atherosclerosis, smoking, symptom, systolic blood pressure, diastolic blood pressure, type of AAD (Stanford), admission of white blood cell, hemoglobin, alanine transaminase, aspartate aminotransferase, albumin, creatinine, and management.

**Table 5:** Results of subgroup analysis and interaction analysis.

Characteristic	No.	OR (95% CI)	*P* for interaction
Age (years)			0.851
<70	1408	1.10 (1.06, 1.14)	
≥70	118	1.12 (0.93, 1.35)	
BMI (kg/m^2^)			0.476
<18.5	57	1.12 (0.97, 1.29)	
≥18.5, <23	445	1.07 (1.02, 1.13)	
≥23	1024	1.12 (1.07, 1.18)	
SBP (mmHg)			0.291
Low (50.00–132.00)	493	1.12 (1.06, 1.19)	
Middle (133.00–157.00)	510	1.10 (1.04, 1.17)	
High (158.00–246.00)	523	1.05 (0.99, 1.11)	
DBP (mmHg)			0.398
Low (26.00–73.00)	480	1.09 (1.04, 1.14)	
Middle (74.00–88.00)	522	1.13 (1.05, 1.22)	
High (89.00–157.00)	524	1.05 (0.98, 1.13)	
WBC (×10^9^/L)			0.593
Low (2.14–9.25)	509	1.07 (0.98, 1.17)	
Middle (9.26–12.48)	508	1.12 (1.05, 1.21)	
High (12.49–29.10)	509	1.08 (1.04, 1.13)	
Hb (g/L)			0.496
Low (11.00–118.00)	508	1.07 (1.02, 1.11)	
Middle (119.00–134.00)	497	1.23 (1.12, 1.35)	
High (135.00–215.00)	521	1.09 (1.03, 1.14)	
ALT (*μ*/L)			0.062
Low (3.00–15.50)	506	1.19 (0.99, 1.42)	
Middle (15.60–28.70)	510	0.89 (0.72, 1.09)	
High (28.80–6817.30)	510	1.11 (1.07, 1.15)	
AST (*μ*/L)			0.969
Low (2.85–17.00)	503	1.09 (0.84, 1.43)	
Middle (17.10–26.20)	512	1.11 (0.95, 1.29)	
High (26.30–11216.00)	511	1.09 (1.05, 1.13)	
ALB (g/L)			0.234
Low (16.10–33.50)	494	1.10 (1.05, 1.15)	
Middle (33.60–37.80)	522	1.19 (1.06, 1.32)	
High (37.90–53.80)	510	1.07 (1.02, 1.13)	
Cr (*μ*mol/L)			0.268
Low (5.00–71.10)	505	1.14 (1.02, 1.28)	
Middle (71.20–97.90)	511	1.01 (0.90, 1.14)	
High (98.00–1718.80)	510	1.08 (1.04, 1.13)	
Gender			0.133
Male	1227	1.12 (1.07, 1.17)	
Female	299	1.05 (0.98, 1.13)	
Symptom			0.055
Chest pain	1166	1.08 (1.04, 1.12)	
Back pain	69	1.11 (0.94, 1.30)	
Abdominal pain	108	1.30 (1.05, 1.60)	
Syncope	19	2.04 (0.79, 5.23)	
Other	164	1.10 (0.98, 1.23)	
Hypertension			0.308
No	469	1.13 (1.06, 1.19)	
Yes	1057	1.08 (1.04, 1.13)	
Diabetes			0.944
No	1468	1.10 (1.06, 1.14)	
Yes	58	1.11 (0.90, 1.37)	
Smoking			0.833
No	1044	1.10 (1.06, 1.14)	
Yes	482	1.11 (1.03, 1.19)	
Stroke			0.805
No	1465	1.10 (1.06, 1.14)	
Yes	61	1.15 (0.85, 1.55)	
Atherosclerosis			0.360
No	1393	1.10 (1.06, 1.14)	
Yes	133	1.23 (0.94, 1.61)	
Type of AAD (Stanford)			0.798
A	703	1.09 (1.04, 1.14)	
B	823	1.08 (1.03, 1.13)	
Management			0.284
Medical	412	1.19 (1.06, 1.34)	
Endovascular	647	1.10 (1.03, 1.17)	
Surgical	467	1.09 (1.03, 1.15)	

Abbreviations: CI, confidence interval; OR, odds ratio; BMI, body mass index; SBP, systolic blood pressure; DBP, diastolic blood pressure; WBC, white blood cell; Hb, hemoglobin; ALT, alanine transaminase; AST, aspartate aminotransferase; ALB, albumin; Cr, creatinine; AAD, acute aortic dissection.

## Data Availability

The data sets used and/or analyzed during the present study were availed by the corresponding author on reasonable request.
